# Atypical parakeratosis in nail unit squamous cell carcinoma

**DOI:** 10.1111/cup.14241

**Published:** 2022-04-25

**Authors:** Aman Prasad, Bridget E. Shields, Yaohui G. Xu, Juliet L. Aylward, Molly A. Hinshaw

**Affiliations:** ^1^ Department of Dermatology University of Wisconsin School of Medicine and Public Health Madison Wisconsin USA

## 1 INTRODUCTION

Cutaneous squamous cell carcinoma (SCC) is the most common malignancy of the nail unit (nuSCC) and presents unique clinicopathologic challenges compared to other locations of SCC for several reasons: (1) clinical similarity to other causes of nail dystrophy such as onychomycosis, chronic trauma, or warts; (2) delayed time to biopsy resulting in missed opportunity to intervene; and/or (3) inadequate biopsy resulting in insufficient specimen for histopathologic examination.^1^, ^2^, ^3^

nuSCC typically presents as lateral onycholysis or as a warty nodule of the nail unit in older men.^1^, ^2^ The etiology is unknown, but infection with oncogenic strains of human papillomavirus (HPV), especially type 16, is a major risk factor for developing nuSCC.^4^, ^5^ Exposure to ionizing radiation and/or chronic nail trauma may also contribute to the pathogenesis.^1^ The main treatment is Mohs surgical excision.^6^

To facilitate diagnosis and optimal management, we conducted a retrospective study of nuSCC at our institution. We also evaluated atypical parakeratosis because in nail clippings and shallow, otherwise non‐diagnostic biopsy specimens, it may be a potentially useful pathologic finding in nuSCC that has not previously been addressed. Atypical parakeratosis is a feature unique to cutaneous SCC compared to warts and psoriasis.^7^ Pap smear data for cervical neoplasia suggest that atypical parakeratosis is an important predictor of progression to SCC in genital mucosa.^8^, ^9^

## 2 MATERIALS AND METHODS

This study was approved by our institutional review board and all data were deidentified prior to analysis. We performed a search of dermatopathology records at our University and the affiliated Veterans Administration Hospital for patients with a histopathologic diagnosis of nuSCC and/or atypical parakeratosis in a nail unit biopsy specimen with or without a final diagnosis of nuSCC. We defined the nail unit as the collection of relevant structures of fingernails or toenails including the proximal and lateral nail folds, cuticle, lunula, nail plate, matrix, nail bed, and hyponychium. We defined atypical parakeratosis as parakeratosis (i.e., retention of nuclei in cornified layer cells) with nuclear atypia (i.e., nuclei which are large, hyperchromatic, and may also have prominent nucleoli). Our search yielded 40 cases dating from 1987 to 2019. Three cases were excluded because no clinical history was available. Five cases were recurrent nuSCC. We reviewed physician notes, photographs, histopathologic slides, and pathological reports for all cases when available.

## 3 RESULTS

**FIGURE 1:**
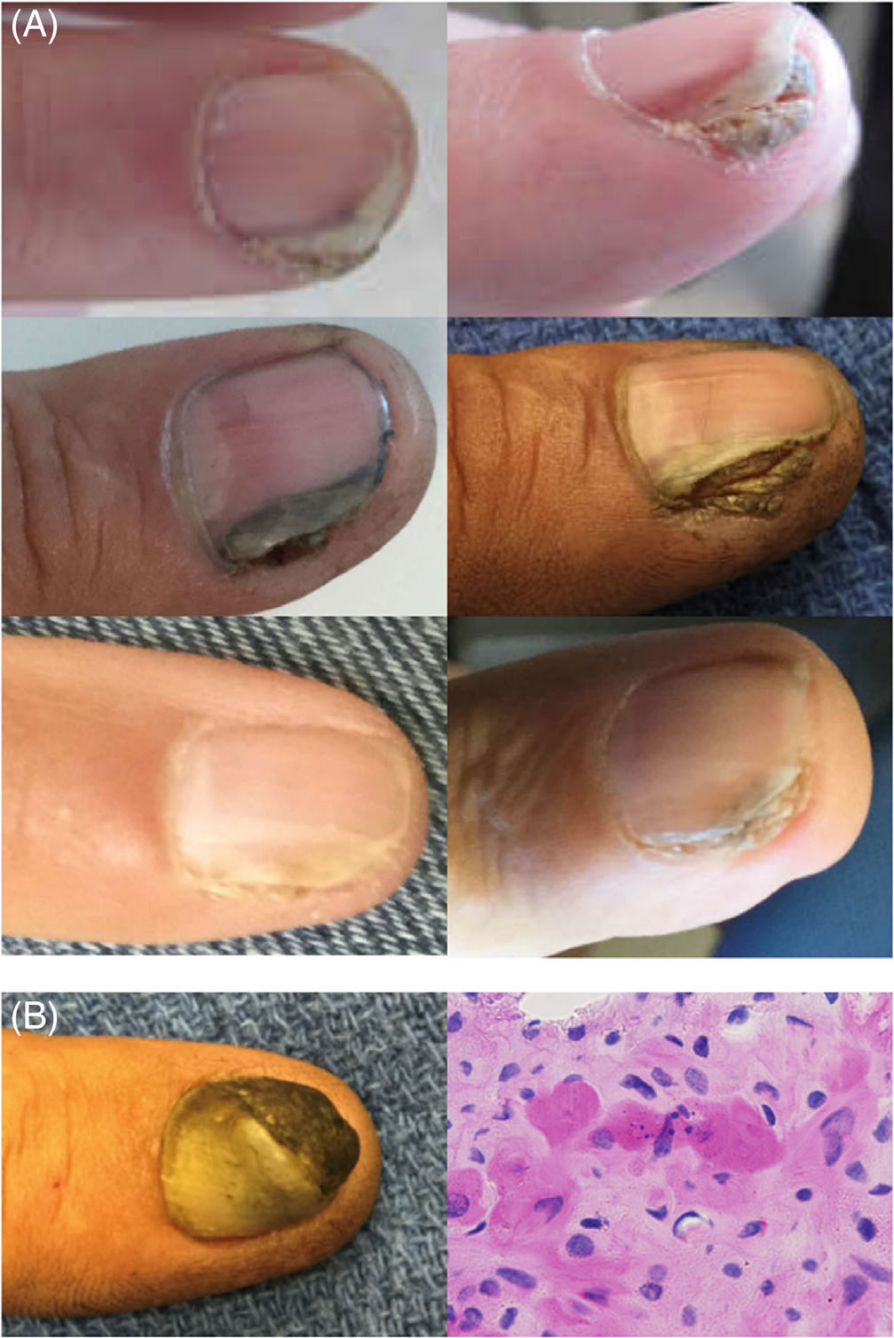
(A) Photographs of nail unit squamous cell carcinoma (nuSCC) for three patients prior to initial biopsy show the common presentation of warty growth with subungual hyperkeratosis and onycholysis. (B) In the lesion depicted, the clinical suspicion was for onychomycosis (left) and only nail plate fragments were obtained on initial biopsy specimen with H&E staining, but this case was later re‐evaluated because of atypical parakeratosis (right, ×80) and eventually a diagnosis of recurrent nuSCC was made

The presenting features of the 32 cases of primary nuSCC are in Table 1. The three most common features for the initial lesion were, in order of frequency, onycholysis, a warty lesion, and subungual hyperkeratosis (Figure 1A). In addition, oozing and pain were frequently reported. The type of biopsy performed was 22 shave biopsies, 4 excisions, and 3 punch biopsies. Twenty‐two biopsy specimens contained epithelium and dermis only, on the other hand, seven contained epithelium, dermis, and plate. The location of the tumor was based on the histopathologic report and was the nail bed in 21 cases, the nail fold in 2 cases, the nail matrix in 2 cases, the nail bed and matrix in 3 cases, and the nail fold and matrix in 1 case. Twenty‐four of the 32 cases were well‐differentiated SCC, 5 were SCC in situ, and 3 were moderately well‐differentiated SCC. All cases were negative for bony involvement.

**TABLE 1:** Patient characteristics

		Percentage
No. of nuSCC cases that were initial diagnoses	32	
No. of nuSCC cases that were recurrences	5	16
No. of patients with nuSCC in multiple digits	2	6
No. of patients with history of other skin SCC	7	22
No. of patients with immunosuppression	4	13
Frequency of atypical parakeratosis where original biopsy specimen was available	15	57
Sex	Male: 23	72
	Female: 9	28
Average age (range)	64.9 y (36–87 y)	
Affected digit	R Thumb: 8	25
	R Index: 4	13
	R Middle: 3	9
	R Ring: 4	13
	R Small: 0	
	L Thumb: 5	15
	L Index: 4	13
	L Middle: 3	9
	L Ring: 1	3
	L Small: 0	
Time to initial diagnosis	57.3 months	

Abbreviations: L, left; nuSCC, nail unit squamous cell carcinoma; R, right; y, years.

A primary motivation of this study was to characterize atypical parakeratosis, which was noted in 15 of the 26 cases for which original biopsy specimen slides (not frozen section slides from the Mohs surgery) were available. Four cases required multiple physician visits and multiple biopsies after an initial visit to eventually reach the diagnosis of nuSCC. Three of these four cases had atypical parakeratosis in the initial biopsy specimen (Figure 1B) but were too superficial to be diagnostic of nuSCC.

Mohs surgery was performed in 29 of 32 primary cases. An average of 2.5 Mohs layers were needed to achieve clearance, the average defect size was 16 × 20 mm, 85% of cases were left to heal by second intention (with grafts and flaps comprising closure in the remaining cases), and recurrence occurred in 3 out of the 29 Mohs cases.

For the five recurrent cancers, the average delay to diagnosis was 54.8 months. One patient had a history of kidney transplant and was on chronic immunosuppression. The primary lesion had been treated by Mohs surgery in the three cases for which treatment data were available, with an average of 2.3 layers to achieve clearance. In addition, the size of the primary tumor in recurrent cases was consistent with non‐recurrent cases. The type of biopsy performed was four shave biopsies and one excision. Three contained epithelium and dermis only, on the other hand, two contained epithelium, dermis, and plate. The location of the tumor was the nail bed in three cases, the nail matrix in one case, and the nail fold and nail bed in one case. Four of the five recurrent cases were of well‐differentiated SCC without invasion to bone or nerve and the fifth case was SCC in situ. Four of the five recurrent cases were noted to have atypical parakeratosis on biopsy specimens at the time of suspected recurrence.

## 4 DISCUSSION

We present a comprehensive retrospective review of 37 primary and recurrent cases of nuSCC at a single, large, academic tertiary care, and transplant center in the United States. Of note, our results suggest that it would be useful for dermatopathologists to evaluate for atypical parakeratosis in nail clippings and in shallow biopsy specimens and comment on its presence as a clue of underlying nuSCC.

Limitations for this study are that it is a retrospective chart review and is not intended to provide correlative statistics for features of nuSCC. In addition, the small sample size, while consistent with the rarity of nuSCC, limits the interpretation and generalizability of our findings. HPV infection is strongly associated with nuSCC, but we were unable to clinically or histopathologically evaluate the association with an HPV infection with high confidence. Finally, the use of the pathology report to identify the location of the tumors is a limitation as the true extent of the tumor could span multiple areas of the nail unit. The paucity of research on nuSCC, including risk factors, presentation, and treatment warrants continued investigation.

## CONFLICT OF INTEREST

The authors declare that there are no conflicts of interest.

## ETHICS STATEMENT

This study was reviewed and approved by the University of Wisconsin Health Sciences institutional review board (approval #2020‐0431‐CP001).

## Data Availability

The data that support the findings of this study are available from the corresponding author upon reasonable request.
